# Editorial: Caregiving and Social Support in the Context of Health and Illness

**DOI:** 10.3389/fpsyg.2020.620357

**Published:** 2020-12-23

**Authors:** Sabrina Cipolletta, Val Morrison, Noa Vilchinsky

**Affiliations:** ^1^Department of General Psychology, University of Padua, Padua, Italy; ^2^School of Psychology, Bangor University, Bangor, United Kingdom; ^3^Department of Psychology, Bar-Ilan University, Ramat Gan, Israel

**Keywords:** caregiver, health, illness, social context, support

Providing unpaid care to a close person (informal caregiving) whether it be for a spouse, a parent, a child or a friend or close neighbor, who are coping with a disability, illness, or frailty, is something that 60% of us may face at some point in our lives (Carers UK, 2019). According to the American National Alliance for Caregiving (2020) family caregivers in 2020 encompass more than one in five Americans. Increased life expectancy and an increased prevalence of chronic disease and comorbidity in the community puts formal care systems under pressure and thus increasingly relies on more informal systems. Some carers offer regular or sporadic support from a distance, others are “on call” 24/7. Many of these carers provide care for many hours a week and for long periods. The role can bring with it challenges, but also opportunities within the context of relationships and adjustment over time. Yet, many questions with regard to these challenges are yet to be answered. For example, what factors are associated with the gains and strains of caregiving? Do caregivers share similar experiences, and are these experiences stable over time? How can informal caregivers be supported? In addition, it seems that caregiving research remains somewhat biased toward care of the elderly or people suffering from dementia, as well as cancer care, and much of it is conducted within western cultures. Thus, much less is known about any unique experiences of caregivers in other contexts and cultures.

We, therefore, decided to put out a call for this special Research Topic for *Frontiers in Psychology* to elicit submissions from a broad range of research and researchers, countries and cultures, perspectives and methodologies, united in that they address the topic of caregiving and support experiences, some of them from a novel conceptual, methodological, or empirical perspective. The 25 papers included here successfully and robustly responded to dual, sometimes triple, peer review, and have surpassed our expectations in terms of both the breadth and depth with which they address our goals. At the time of writing, this Research Topic has already elicited over 47,000 views on Frontiers. This high number reflects scientific interest in the issue of caregiving and strengthens our call for further research in this field.

The data presented within the papers are drawn from *diverse populations of carers*. These include adult caregivers vs. child caregivers; informal vs. formal caregivers, and care provided within families by spouses, parents, or adult children. This diversity highlights aspects of role expectations and care norms (e.g., Pertl et al.; Morrison and Williams) as well as the relational aspects (e.g., Cardinali et al.; Kroemeke Sobczyk-Kruszelnicka) and influences within and across generations (Zarychta et al.). The studies also focus on *different illnesses* amongst the care recipients (stroke, heart disease, chronic pain, liver disease, cancer, arthritis to name but a few). Whilst many common characteristics of care needs and associated care tasks can be seen, there can be illness specific implications for the responses of caregivers and the care required. Consider, for example, the needs and worries of the caregiver of an adult who is waiting for a liver transplant (Cipolletta et al.) compared to those of the caregiver of a child having had heart surgery (Vainberg et al.), or the care needs of a person facing infertility treatment with potential positive outcomes (Malina et al.) compared to the needs of someone with a progressive illness (Avargues-Navarro et al.).

In addition, care needs can be influenced by whether an illness is common and possibly better understood than a rare condition (as reported by Cardinali et al.). Interestingly, Otero et al. compare across a wide range of illnesses in relation to the concurrent association between illness type and caregiver distress and revealed little differences. Future studies could usefully explore such associations by taking into account the length of time spent in caregiving and also different trajectories of illness because caregiving and responses to it are not static, as exemplified in the qualitative and quantitative longitudinal data included in this Topic. Cornelius et al. show that caregivers can be affected as early as at the point of exposure to the initial symptoms of a cardiac event among their loved ones. Horn et al. highlight the additional issues facing the caregivers of those with multimorbidities as opposed to caregivers with a unitary condition. All in all, the context of caregiving and the characteristics of both the caregiver and care recipient are important considerations.

The current collection sheds light on the critical question of whether caregivers' responses impact *patients' experience*. Mohammadi et al. detected less catastrophizing and reduced pain behaviors amongst patients who had carers who engaged in distracting behavior, with the converse also holding. Kroemeke's study of the role of protective buffering revealed that it is not inevitably associated with poor adjustment as previously considered. Moreover, according to the dyadic approach, the trajectory of influence is not only unidirectional, from the caregiver to the patient, but there is evidence that patients and caregivers impact each other, simultaneously. From studies that applied a dyadic analysis, we learn how important it is to consider caregiving within its relational context (Cipolletta et al.; Horn et al.).

Interestingly, only four studies in this collection have specifically focused on social support in the caregiving context. As in numerous former studies, the beneficial effect of supportive social interactions on caregivers has been detected among parents of children with rare diseases (Cardinali et al.) as well as among partners going through fertility treatments (Malina et al.). Yet, in Cipolletta et al.'s study, perceived social support did not predict patients' and caregivers' psychological symptoms, and Kroemeke and Sobczyk-Kruszelnicka detected, somewhat surprisingly, a beneficial effect for a protective buffering type of support. We still, however, need more studies to fully understand the roles different forms of social support play in the often changing context of caregiving and in relation to different outcomes.

We were keen to include papers that observed caregiving through “new eyes.” For example, Fernández-Ballesteros et al. claim that paternalism, despite its negative connotation, is not necessarily an inappropriate reaction toward the elderly. Paternalism, it is proposed, can be beneficial as long as it is contextualized and as long as caregivers demonstrate protection but not overprotection toward their care receivers. In another example, whilst the concept of caregiver burden has been extensively studied previously our topic offers several papers that consider caregiver burden from an “occupational burnout” perspective. Applying the perspective of occupational burnout is novel in its consideration of balancing role characteristics (personal, relational, and contextual) with accomplishments and taking a more detached “professional” approach to the role with caregivers, i.e., perceiving it more as a workload rather than a personal matter. The occupational perspective can be specific to formal caregivers (Gérain and Zech) and those with dual roles of caregiver and employee (Converso et al.), or housewives acting also as family caregivers (Avargues-Navarro et al.). Finally, Bei et al. shed light on a less studied area of caregiving, namely that of being a distance caregiver which can pose a major difficulty to many informal carers (potentially even more so in the context of the SARS-CoV 2 global pandemic).

As well as addressing a broad range of conditions and contexts, this collection of studies represents those employing a wide range of *different designs, methods, and analyses*, with innovative (including with photograph elicitation, intensive diary methods, longitudinal case study), as well as traditional research methods exemplified (surveys, interviews, experimental methods, and intervention studies). Important across all research fields is an understanding of how concepts are defined and measured, and to that end, the study by Aubeeluck et al. makes a valued contribution to the topic by presenting a validation study of a new assessment of the quality of life in Huntington's Disease. From national surveys (Converso et al.; Otero et al.; Haugland et al.; Yang and Zheng) we obtain epidemiological knowledge that may be useful to planning healthcare services or care policy. Longitudinal studies (Pertl et al.) allow the effects of caregiving on caregivers' well-being to be evaluated, and from qualitative studies, some also longitudinal (Cardinali et al.; Freda et al.; Morrison and Williams; Vainberg et al.) we gain an in-depth understanding of caregivers' fluctuating experience and critically, their motivations. Finally, experimental studies allow us to test hypotheses regarding the effectiveness of interventions in a controlled situation. Hasuo et al. conduct a randomized controlled trial to evaluate the benefit of heart rate biofeedback to sleep amongst cancer caregivers, and Rasmus and Orłowska, conducts a controlled evaluation of group therapy to stroke caregivers who cope with their care recipients' communication deficits as a result of aphasia. More efforts should be channeled toward developing interventions to help caregivers cope. Psychological interventions were found to be successful in reducing the burden on informal caregivers and their mental stress, at least in the context of stroke, as presented in a review by Panzeri et al. Finally, potentially taking the intervention field forward, Petrovic and Gaggioli present a scoping review of the emerging field of digital mental health tools that could offer innovative means of supporting caregivers' needs.

It is hoped that this special Research Topic on caregiving, whilst broad and multi-faceted, will be received as a meaningful representation of the ways in which this critical topic, which is relevant to all of society and all societies, is being addressed across Europe and beyond.

The conclusions of each of the 25 papers contained within this collection point us to a need for more research and tailored intervention. In particular, the differences between formal and informal caregivers in terms of the emotional consequences of caregiving are not straightforward and deserve more research- including, for example, a study of where informal caregivers share their role with formal paid carers. There remains a need for more studies comparing male and female caregivers, given the cultural and generational differences in gendered expectations. More attention needs to be paid to the impact on young caregivers, often those with multiple roles, and there is a pressing need to consider caregiving in the context of different illness and treatment trajectories. We propose that caregiving research should embrace a broader perspective that looks at individuals in context and which takes the perspective of a multiplicity of actors such as extended families, educators, and employers, into account. In addition, the financial burden of caregiving needs to be addressed at a systematic level and interdisciplinarity represents a new frontier for the studies on caregiving.

Rather than providing any definite solutions or answers to the challenges of informal caregiving that need to be faced by our aging society, we present this Research Topic as a means of highlighting the diversity of research and raising further questions. We particularly encourage the sharing of such thoughts with other professionals. Combining knowledge from different disciplines to think out of the box will better enable the identification of innovative and supportive solutions to caregiver needs.

## Author Contributions

SC, VM, and NV conceptualized the topic and wrote the editorial. All authors contributed to the article and approved the submitted version.

## Conflict of Interest

The authors declare that the research was conducted in the absence of any commercial or financial relationships that could be construed as a potential conflict of interest.

## References

[B1] American National Alliance for Caregiving (2020). Caregiving in the U.S. 2020. Available online at: https://www.caregiving.org/caregiving-in-the-us-2020 (accessed December 13, 2020).

[B2] Carers UK (2019). Policy Briefing, Facts About Carers. Available online at: https://www.carersuk.org/for-professionals/policy/policy-library (accessed December 13, 2020).

